# New Aspects in the Formulation of Drugs Based on Three Case Studies

**DOI:** 10.3390/molecules21050577

**Published:** 2016-04-30

**Authors:** Patrick Frohberg, Thi Nhat Phuong Nguyen, Joachim Ulrich

**Affiliations:** Thermische Verfahrenstechnik, Zentrum für Ingenieurwissenschaften, Martin-Luther-Universität Halle-Wittenberg, Zentrum für Ingenieurwissenschaften, Halle 06099, Germany; thi.nguyen@iw.uni-halle.de (T.N.P.N.); joachim.ulrich@iw.uni-halle.de (J.U.)

**Keywords:** *in situ* coating, freeze casting, biocomposites, proteins, controlled release

## Abstract

The improvement of pharmaceutical dosage forms, such as tablets, towards drug delivery control and cost efficiency is of great importance in formulation technologies. Here, three examples: *in situ* coating, freeze casting and protein-based biocomposites are presented that address the above mentioned issues and contribute to further developments in formulation technologies. The *in situ* coating increases the economic efficiency by saving process steps in comparison to a conventional tableting process and provides a crystalline coating for a tailorable drug delivery rate. The freeze casting allows the control over the surface area of a drug delivery system (DDS) by providing different numbers and sizes of pores, which in conjunction with adequate additives offer an efficient instrument for drug delivery control, especially by accelerating the dissolution effect. Protein-based biocomposites are attractive materials for biomedical and pharmaceutical applications that can be applied as a polymeric DDS. They inherently combine degradability *in vivo* and *in vitro*, show a good biocompatibility, offer sites of adhesion for cells and may additionally be used to release embedded bioactive molecules. Here, a new approach regarding the incorporation of crystalline active pharmaceutical ingredients (API) into a protein matrix in one process step is presented. All three presented techniques mark decisive progress towards tailor-made drug delivery systems with respect to function, economic efficiency and the generation of additional values.

## 1. Introduction

Active pharmaceutical ingredients need to be dosed properly with respect to time and concentration and location in the human body. Additionally, the DDS needs to satisfy economic demands that are eventually reflected in an acceptable pricing.

Novel production techniques and the related drug delivery systems crucially contribute to the solution of the addressed issues. Therefore, three novelties in the field of advanced drug delivery systems and processing technologies are introduced. These include self-coated tablets produced via an *in situ* coating process that provide a crystalline instead of an amorphous coating. Furthermore, the application of the freeze casting technique allows us to control the dissolution rate of drugs through the provided surface area within a tablet. Finally, protein-based biocomposites as controlled drug delivery systems are introduced. This involves biochemical approaches and a novel reactive processing technique capable of a cost-efficient production of protein-based delivery systems that offer key benefits, such as non-toxicity, biodegradability, edibility and biocompatibility for diverse applications in tissue engineering, drug delivery and gene therapy. The variety of available protein sources, mild processing conditions and tailor-made properties facilitate proteins as raw materials for biocomposites with incorporated active ingredients that provide a controllable release functionality.

The objectives of this study are the introduction and discussion of new technologies that can address the traditional problems in the formulation of drugs. Therefore, three case studies are presented and summarized that highlight the advantages of the respective product and process design substantiated with concrete examples and methodologies.

## 2. *In Situ* Coating

The *in situ* coating process contributes to a reduction in the number of process steps in conventional tableting and thus significantly increases the cost-efficiency of the whole production process [1,2,3,4]. The *in situ* coating technique is based on the utilization of the traditional melt crystallization process that offers the potential to separate substances. Originating from a molten drop, the melt crystallization process initiates the coating process, which consists of the component that crystallizes first on the drop surface after reaching the surface of a cooled steel surface. Thereby, the other component, e.g., the API is enclosed by the coated drop.

**Figure 1:**
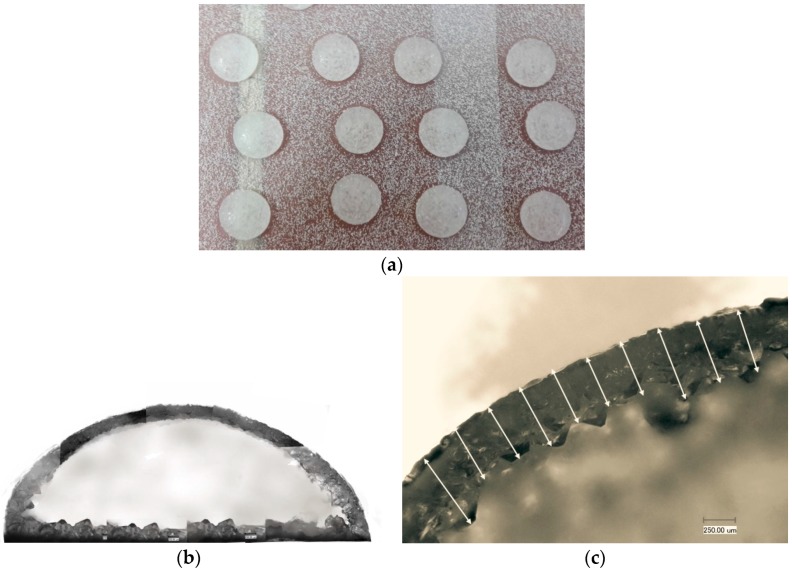
(**a**) Drop formed pastilles with coated active pharmaceutical ingredients; (**b**) Crystalline coat of the pastilles (cross section); (**c**) Layer thickness measurement of a pastille cross section.

**Figure 2:**
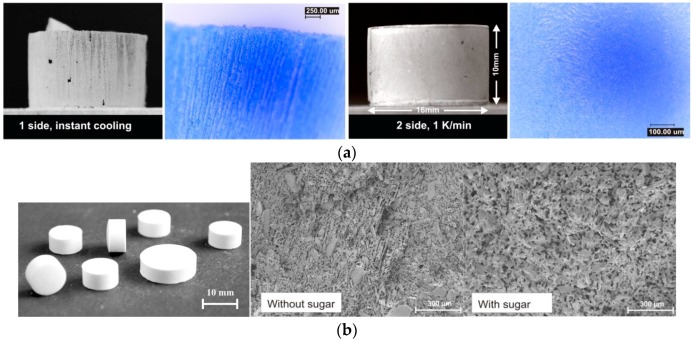
Examples of micropore structures of freeze-casted tablets: (**a**) needle-like structure (left) and planar lamella structure of freeze casted cocoa tablets [13]; (**b**) microspore structures of freeze-casted paracetamol tablets, with and without additive (*i.e.*, sugar-sucrose) [13].

Figure 1 shows drop-formed pastilles on a cooled steel surface including seed particles to induce nucleation on the pastilles. The crystalline coats are presented in Figure 2 as a cross section. The crystalline coatings, presented in Figure 1b,c need to be thick enough to provide a sufficient mechanical strength for handling and sealing the incorporated API. In order to apply the *in situ* coating process, the coating material has to meet the following requirements:

- Mixability with the drug material in the melt
- Crystallization prior to the drug material
- Stability at its melting point, and stability at the reduced melting temperature of the mixture with the API.

Thereby, the main benefit of the *in situ* is the avoidance of a separate tableting and coating process step, because, after mixing both, the coating material and the API will be molten and form drops. This will be followed by autonomously occurring process steps. These involve the formation of a crystalline coating by positioning the molten drop on a cooled surface. Thereby, the coating material crystallizes inwards towards the drop and exists either as eutectic mixture of the components or, if the API is liquid at ambient conditions, as a liquid core of the eutectic mixture.

By utilizing a conventional steel belt technology with drop forming materials, a multitude of self-coating drops can be produced simultaneously. However, in the case of a high viscosity of the drop-forming mixture, a seeding procedure, e.g., a powder bed [5,6] (see Figure 1a) or an ultrasound radiation [6] is required. Both are known techniques aiming to ensure that all drops start nucleating at their surfaces at the same time. This ensures uniform thicknesses of the pastille coatings, as presented in Figure 2b,c. The technology and the proof of concept are available and described in [7,8].

## 3. Freeze Casting—An Alternative Technique to Produce Controlled Release Tablets

A controlled drug release from a DDS is of high importance in order to avoid numerous consequences of drug delivery issues for the patient. To ensure the effectiveness of the treatments, the concentration profiles of the drug substances are required to follow strict standards. Several techniques, namely multilayer tableting and coatings with functional membranes, are applied. Unfortunately, these methods are complex and expensive. A simpler way to modify the dissolution or the dispersion of drug substances without changing the chemical composition of the DDS is to control the contact surface areas between solid and liquid phases. To achieve a higher contact surface area, drug substances are often used in fine powder forms. However, they tend to agglomeration, which often occurs during the usage as well as during the manufacturing process [9]. To solve this issue, a porous tablet tailored by the freeze casting process represents a new approach [9,10,11,12,13,14].

Presently, the freeze casting technology is reported as a potential method to produce tablets with controllable release [9,11,12,13]. Thereby, the dissolution behavior of tablets is adjusted based on two main factors: (1) the contact surface area; and (2) the effect of additives, such as disintegrants or binders.

This method is based on the crystallization of solvents. In almost all cases, water is used to generate tailored porous microstructural tablets. The production process can be described as follows: An aqueous liquid suspension of the relevant substances is frozen in a form-giving tool called mold and, subsequently, the ice (the frozen liquid of the suspension) is removed by a sublimation or an evaporation process. Due to the volume expansion upon solidification of water, a cold compression of the systems occurs. Thus, after removing ice crystals, a porous solid body in the desired mold form is achieved without any external pressure. By this technique, the pore morphologies are considered as the negative images of the crystals formed by freezing the liquid of the former suspension. This means that the initial ice crystals determine the structure of the pores in solid bodies in terms of size, shape, amount and distribution [13]. Therefore, by a proper control of the crystallization of the liquid phase, pore structures as well as porosities of produced solid tablets can be adjusted. This allows the tailoring of the contact surface area between the liquid and solid phase when the porous tablets are in contact with a liquid media. As a result, the dissolution/dispersal rate of the DDS and, consequently, the API release is controlled.

The most critical challenge is the determination of the suitable porosity. The contact surface area is determined by the geometry of pores, which include the size and the shape of individual pores. Furthermore, the contact surface is also influenced by the distribution of pores inside the solid body [13]. “Are the pores open or closed? Are they separated or connected?” are key questions. In general, the contact surface area becomes larger with smaller sizes of pores and a higher number of pores. It is noted that the size of pores generates, unfortunately, the opposite influences on the dissolution and the diffusion processes. For a fast diffusion process, pores of maximum sizes are required. In contrast to this, the pore sizes need to be minimized to achieve a larger surface area, which lead to a faster dissolution. Thus, a certain critical pore size exists, at which the diffusion process is not disrupted or slowed down. Although the determination of the critical pore size is of high importance, up to now no practical method is available. In 2012, Pradzynski *et al.* [15] found that the smallest ice crystals (~0.13 µm), which can be formed are about 475 times of the size of a water molecule. In fact, this value is just a reference value that gives an idea, how small the pore size in theory could be. In practice, the ice crystallization is more complicated in suspension systems. Furthermore, the final pore size is also affected by the distribution of solid particles during the solidification determined by the solid loading, solid particles size as well as their own connections. Applied in ceramic materials, a large distribution of pore sizes in a range of 2–200 µm was found [16]. Recently, a pore size range of 2–10 µm was found in practical studies in pharmaceuticals and food systems [12,13].

Previous studies demonstrate that this technique can be applied to a wide range of solid materials in the field of pharmaceuticals [12,17,18,19,20,21], foods [9,10,11,13] and biomaterials [21]. The porosity is adjustable from very dense, less than 10% [22], to highly porous, up to 75% [9,13] and an average value of about 50%–60% can easily be achieved in many cases [11,12,13,19,21,23]. The pore structure can be quite different from needle channel to planar lamella structure, as presented in Figure 2.

In order to adjust the pore morphology as well as the dissolution behavior of freeze-casted tablets, a full control of a solidification process during freezing is required. All the factors that affect the crystallization or solidification of the water and suspension need to be considered. This includes the operating conditions, such as freezing temperature and freezing time, the physical properties of the solids, the composition of initial suspensions (solid loading) and the additives.

The effect of the operating conditions on the behavior of the pore formation is identical and clearly understood in different systems. The general rules were found and are presented in literature [16,24]. It was also reported that the operating conditions upon freezing are significantly important in designing the pore volume of produced solid tablets. However, in the pharmaceutical and food industry, the incorporation of a functional additive is often crucial to reach the requirements in terms of both dissolution and mechanical strength of tablets.

Thereby, the additives should dissolve in liquid media of suspensions. They are able to alter the thermodynamic properties of the aqueous phase and, therefore, lead to a different crystallization behavior of the suspensions. In addition, when an additive has a binding function, it will crystallize after the freezing process or solidify itself and stays in between the solid particles of the main solid active ingredient. The matrix of local bridges of a binding-additive stabilizes the porous structure of the freeze-casted solid bodies and, therefore, the mechanical strength of produced tablets is increased.

**Figure 3:**
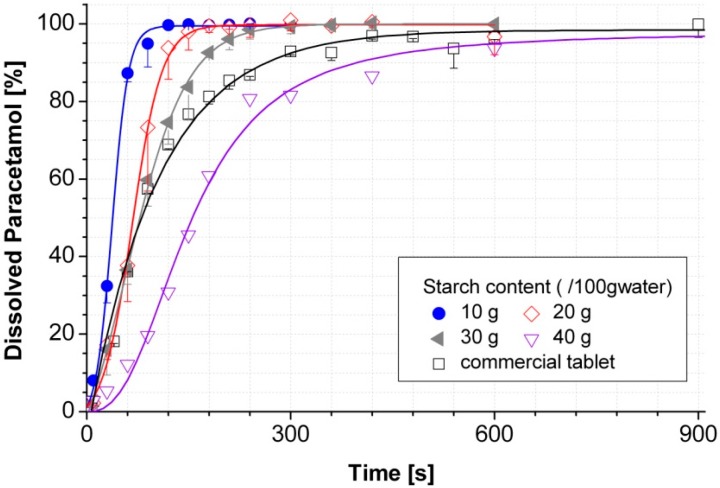
The dissolution profile of freeze casted paracetamol tablets in function of the modified starch contents [13].

By a proper selection of the additives based on their hydrophilicity, solubility, dissolution rate and permeability, a desirable dissolution or dispersion profile and mechanical strength of tablets can be tailored. For example, by addition of easily water-soluble sugar alcohols as additives, fast dissolving cocoa tablets were successfully produced. Thereby, the fast release of the binding agents facilitates an instant dispersion of the tablet, when it is dropped into a liquid. A manageable freeze-casted tablet with a dissolving time of less than 1 min was easily and reproducibly obtained. The technology and the proof of concept were presented by Nguyen and Ulrich in 2013–2015 [9,11,13]. Silica sol (Bindzil) was found to be a very effective binder; however, it also slowed down the release of drugs, because of its strong binding function [19]. In other case studies [12,13,14], modified starch as additive was successfully used. Its content was found to be the main factor in control of the release rate in paracetamol freeze casted tablets. As shown in Figure 3, by a variation of the modified starch content between 0.10 and 0.40 g/g water, the time required to release 100% paracetamol from the tablets was prolonged from 2 to more than 15 minutes. In particular, recent studies [11,12] demonstrated that sugar alcohols, especially sucrose, cause an alteration of the pore morphology. Instead of needle-like channel structures, a honey-comb-like structure was found in thecase of sucrose as additive (see Figure 2) [11,12].

Based on the reported studies, is was demonstrated that the freeze casting technique can be successfully applied to produce controlled release tablets. Freeze-casted tablets are able to compete with commercial compressed tablets in terms of both the dissolution/dispersal time and the mechanical strength [11,12]. The advantage of the freeze casting technique is not only the proper control of the pore structure, but also low operating temperatures without addition of external pressure. This technique is especially reliable for heat and sensitive active ingredients, which are not able to be tableted by conventional compression processes. Furthermore, the freeze-casted products provide a prolonged shelf-life due to the low temperature processing and the low moisture content of the product. Therefore, it is a promising alternative method for the processing of sensitive active pharmaceutical ingredients, proteins and enzymes.

## 4. Protein-Based Biocomposites as Drug Delivery Systems

**Figure 4:**
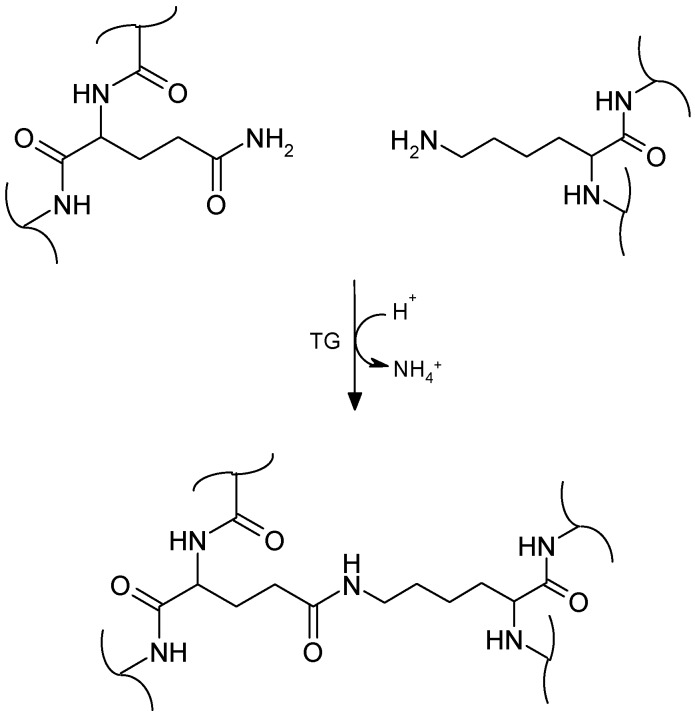
Reaction mechanism of mTG: formation of covalent ε-(γ-glutamyl-)lysine bonds.

Proteins are biological macromolecules consisting of one or more long chains of amino acid residues, whereby, commonly 20 kinds of side chains (R groups) can be found in proteins that vary in size, shape, charge, hydrogen bonding capacity, hydrophobic character and chemical reactivity. This molecular diversity holds a considerable potential for the formation of linkages that differ with respect to their potential reactivity to cross-link and thus, contribute to the large range of protein properties [25,26]. The cross-linking of proteins refers to the formation of covalent bonds between polypeptide chains and can be catalyzed, e.g., by enzymes such as microbial transglutaminase that catalyze acyl-transfer reactions between a γ-carboxyamine group of a peptide or protein-bound glutamyl residue and a primary amino group of various substrates including the ε-amino group of lysine or lysyl residues in proteins as shown in Figure 4 [27,28].

A variety of plant and animal proteins have been considered as raw materials for the production of biobased polymers [29,30]. The diversity of protein-based products has undergone a profound recent expansion, especially towards the development of novel multifunctional biomaterials and biocomposites. Among them are gels, foams, fibers, blends and composites, mainly applied for biomedical applications such as scaffolds and biomimetic materials for tissue regeneration, drug delivery systems and biosensors [31,32]. Among them are protein-based biocomposites consisting of proteins or protein blends and additives or fillers that are incorporated into the protein (blend) matrix. Depending on the particle size of the incorporated additives, bionanocomposites, biomicrocomposites and biomacrocomposites can be distinguished [33,34,35]. Various embedded organic and inorganic fillers in the form of loose or coherent particles or fibers contribute to the enhancement of physical properties and to the reduction of costs of protein-based biocomposites [35,36].

In general, proteins are non-toxic, biodegradable, compostable and biocompatible and, therewith, highly suitable raw materials, e.g., for polymeric carrier and release systems in biomedical or pharmaceutical applications. Protein carrier materials for applications such as drug delivery systems in the form of films, sheets, moldings (including capsules), coatings, gels, *etc.,* can be obtained by the formation of a relatively organized three-dimensional macromolecular network, stabilized by hydrogen bonds, hydrophobic interactions and disulfide bonds through solvent casting or thermoplastic processing methods [29,37,38]. Both methods allow an effective incorporation of additives such as active pharmaceutical ingredients. However, only the thermoplastic processing of proteins facilitates highly efficient manufacturing methods with commercial potential for a large-scale production. Existing plastic processing machinery, including thermoforming, various types of injection molding, compression molding, extrusion (films and sheets) and extrusion coating and lamination have been used for the production of protein-based biopolymers [39,40,41,42].

**Figure 5:**
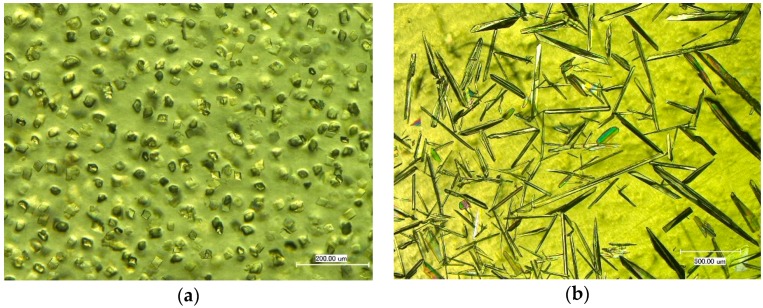
Light microscopy exposures of biocomposites (protein: sodium caseinate) with incorporated crystalline potassium nitrate ((**a**) trigonal phase-III KNO_3_; and (**b**) orthorhombic phase-II KNO_3_).

A new concept for the incorporation of functional crystalline additives by an inner-material crystallization was introduced by Frohberg *et al.* [43]. The protein and the additives are dispersed and solubilized in a solvent (e.g., water or ethanol) followed by solvent removal (drying step). By spreading the protein solution on a casting or product surface and allowing the solvent to evaporate, films, coatings and moldings can be produced and the dissolved additives crystallize inside the protein matrix [29,43,44]. Following this approach, Stolte *et al.* [44] investigated the influence of crystal size and shape of potassium nitrate in protein materials (see Figure 5) on the physical properties of the biocomposites. The crystal habit, size and their distribution were found to have a profound influence on the mechanical properties and the dissolution and release behavior of the additive.

Frohberg [37] successfully produced biocomposites with crystalline additives for controlled delivery systems by continuous reactive extrusion processing. Thereby, an enzymatic cross-linking reaction aims at the direct modification of the protein structure, thus, enables the control of the biocomposites physical properties. This approach combines the thermoplastic processing of proteins with the incorporation of functional additives, the enzymatic protein cross-linking and the shaping of protein-based biocomposites by conventional large-scale twin screw extrusion [37].

In protein-based biocomposites, the crystallization of additives serves both the incorporation of the additive for a controllable carrier and release functionality and the tailoring and optimization of the material properties. An appropriate process design allows the control of the crystal growth and assures a defined mean crystal size [37]. Secondary additives contribute to a specific crystal habit by inhibiting the growth of specific crystal faces [44]. The suitability of protein-based biocomposites as drug carrier and delivery system additionally depends on the barrier properties and the solubility. The barrier properties of a protein material are related to the nature and density of the macromolecular network and depend, more particularly, on the proportion and distribution of polar and non-polar amino acids. Generally, protein materials have a high water vapor permeability and a low permeability to non-polar substances such as oxygen, nitrogen, aromas and oils [29,30,45]. Intermolecular interactions, however, determine the solubility of the protein-based biocomposites. The natural presence or introduction of covalent intermolecular bonds (e.g., through enzymatic cross-linking) and/or a high interaction density lead to protein materials that are partially or even completely insoluble in water [29,46]. Therewith, the enzymatic cross-linking is one product design tool to control the solubility and, thus, the release of the active ingredients from the protein matrix.

The active ingredient release from a protein-based polymer is a complex process of solvent absorption, polymer swelling and/or disintegration followed by the dissolution of active ingredients and its diffusion into the surrounding environment. Therewith, numerous opportunities for a controlled drug release are available that can be implemented by a proper product and process design.

Proteins are highly suitable raw materials with considerable application potential in the field of drug delivery systems. The variety of protein sources and their complexities contribute to protein materials with unique functional properties that can be additionally tailored and optimized by product and process design approaches. Additionally, protein-specific aspects such as edibility and biocompatibility predestine protein-based biocomposites for applications in biomedicine and pharmacy.

## 5. Conclusions

It has been shown that besides of all existing technologies in the formulation of drugs, new technologies are under development that promise drug delivery systems with advanced and tailorable release properties that can be manufactured in very cost-efficient ways. The three examples presented in this study clearly outlined these aspects, the key benefits of which are presented in Table 1.

By applying the *in situ* coating technique, process steps can be saved, leading to a more economical production of drug delivery systems. The freeze casting technique offers a highly controllable drug release through adjustments of the contact surface area and the effect of incorporated additives. Additionally, the low operating temperatures of this technology allow the processing of heat sensitive active ingredients. Protein-based biocomposites are highly versatile drug delivery systems with adjustable physical properties. Embedded in a continuous large-scale production process, active pharmaceutical ingredients are incorporated by crystallization and the properties of the biocomposites are controlled by enzymatic cross-linking of the protein matrix. The additive release is controlled by product and process design approaches.

The three case studies reveal new and advanced techniques for the economical production of drug delivery systems that offer unique features over conventional tableting process. Substantiated with the proof of concept, each of the presented studies address one or more problems in the formulation of drugs, ranging from economic advantages due to the saving of process steps to highly controllable and biodegradable drug delivery systems. Therewith, the foundation for an industrial implementation of these techniques for existing problematic areas was laid.

## Figures and Tables

**Table 1:** Summary of positive points of aforementioned new technologies in the formulation of drugs.

New Technologies	Cost Reduction through Less Process Steps	Better Drug Delivery Control by	Improved Product Properties by
*In situ* coating	shaping crystallization coating	crystalline coats	crystalline coats
Freeze casting	compressing surface area creation additive dosing	controllable surface areas and additives	low temperature and non-external pressure processing
Protein-based biocomposites	continuous reactive extrusion processing, including crystallization and shaping	carrier and release systems with controllable physical properties	cross-linked and tailored protein matrix
